# Rehabilitation Protocol for Lunate Fracture in a Clinical Case Report

**DOI:** 10.7759/cureus.61892

**Published:** 2024-06-07

**Authors:** Chitwan S Agrawal, Mitushi Deshmukh, Sakshi Padmawar

**Affiliations:** 1 Department of Musculoskeletal Physiotherapy, Ravi Nair Physiotherapy College, Datta Meghe Institute of Higher Education and Research (Deemed to be University), Wardha, IND

**Keywords:** kienböck's disease, physiotherapy, wrist fracture, lunate fracture, rehabilitation

## Abstract

A carpal injury called a lunate fracture can cause severe carpal instability if treatment is not received. After the scaphoid, triquetrum, and trapezium, the lunate is the fourth most frequently fractured carpal bone. Due to lunate fracture, the functional prognosis is uncertain, and conservative treatment frequently results in surgery. Lunate fracture may be caused by anatomical features such as ulnar and radial variation, although necrosis is not commonly one of them. Vascularization in the lunate is unstable and dependent on the capsular arterioles. High shear stresses are frequently applied to the lunate, which rubs against the triangular fibrocartilage complex and the radius. This could lead to a fracture. Intraosseous compartment syndrome is most likely caused by the inflammatory condition Kienböck's disease. In order to protect the lunate against shear and compression loads, surgery entails decompressing the lunate. The consequences of current osteotomy techniques on biomechanics will be discussed. While some osteotomies may lessen the stresses conveyed to the lunate, they may also put too much strain on the ulnar side of the lunate. Techniques for treating wrist degeneration comprise extra-articular methods that keep the mid-carpal joint's architecture and vascularization intact. Bone grafting or repair may be indicated if lunate destruction is extensive. In the last phases, palliative methods are employed. For Kienböck's disease, there are currently no efficient biological therapies. The lunate is prevented from collapsing by decompression osteotomies, giving time for natural healing. The patient came to Acharya Vinoba Bhave Rural Hospital Outpatient Department of Orthopedics with a complaint of pain in her right wrist. At a private hospital in Amravati, the patient had gone through a proximal row carpectomy on the right side. After undergoing post-operative physiotherapy, the patient showed good improvement in her functional activities and quality of life. Physiotherapy helps in gaining back the functional activities for the post-operated lunate fracture patient.

## Introduction

The proximal hyperemic section of the joint is removed during a modified arthroplasty procedure for limited degenerative arthritis of the distal radioulnar joint, leaving the unaffected distal region intact [1]. Depending on the cubito-dorsal artery, a branch of the ulnar artery, the pisiform bone is vascularized. This procedure attempts to lessen discomfort, replace missing bones, maintain the space between the radius and capitatum and the scaphoid and triquetrum, and avoid carpal collapse. The vascularized relocation, which is suggested at stage III of conventional classifications, is verified using X-rays and MRI. The method entails employing ligament reconstructions to stabilize the transferred bone and may also include radiation shortening or scaphocapitate-restricted arthrodesis to lessen stress [2]. The "compromised" wrist's secondary consequences of the falling lunate include the following: at the radiolunate and mid-carpal articulations, the central column is deteriorating; the middle column's collapse; radial column collapse; proximal row instability; and, lastly, radial column degeneration [3]. Lunate fractures result in irregularities on the articular surfaces. The outcome is good if the fracture is repaired in a stable form. However, there is a chance that the central column will degenerate if there is secondary degeneration of the lunate facet and capitate with each other [4,5]. With a scaphocapitate fusion, the central column can be avoided because it cannot be rebuilt. On rare occasions, the capitate articular surface is unharmed, allowing for hemiarthroplasty [6,7]. Surgery known as limited wrist fusions is performed on individuals with carpus-related arthritis to reduce pain and maintain wrist motion. With the main objective of fusing damaged joints while preserving unaffected movement, the decision about motion-preserving surgery depends on the origin of the arthritis, involved joints, and spared joints. It is typical for the radial column to collapse in conjunction with central column degeneration or collapse [8,9]. If avascular fragments are shown to be the cause of synovitis or substantial widening on a sagittal CT scan, the lunate is excised. The surgeon must use caution to prevent ulnar translocation by not injuring the volar carpal ligaments, which are connected to the volar pieces [3]. The Sauvé-Kapandji treatment can treat ulnar impaction syndrome cases, enhance mobility in the distal radioulnar joint, and lessen pain. It works best for painful and limited forearm rotation following a distal radius fracture, together with an old dislocation or joint damage [10]. In order to restore function following the excision of the distal ulna, the radioulnar joint fusion technique is a rather uncommon treatment for patients with distal radioulnar joint instability. The treatment is frequently done after other attempts to maintain forearm rotation have failed, and the fusions frequently necessitate additional surgery to establish a union. To hasten healing, iliac bone grafts are frequently incorporated into the fusion region today [11].

## Case presentation

Patient information

The patient was a 67-year-old female who was referred for physiotherapy with a primary complaint of pain in her right wrist. The patient states that she fell off a tractor and broke her right wrist. Pain is sudden in onset and severe in nature; it is non-radiating, shows no diurnal variation, and is non-progressive in nature. The patient gives an alleged history of slip and fall four years back sustaining an injury to her right wrist. At a private hospital in Amravati, the patient had gone through a proximal row carpectomy on the right side. Table 1 shows the timeline.

**Table 1:** Timeline

Episode	Date of episode
Date of admission	15/08/2023
Date of operation	17/08/2023
Date of examination	22/08/2023

Clinical findings

**Figure 1:**
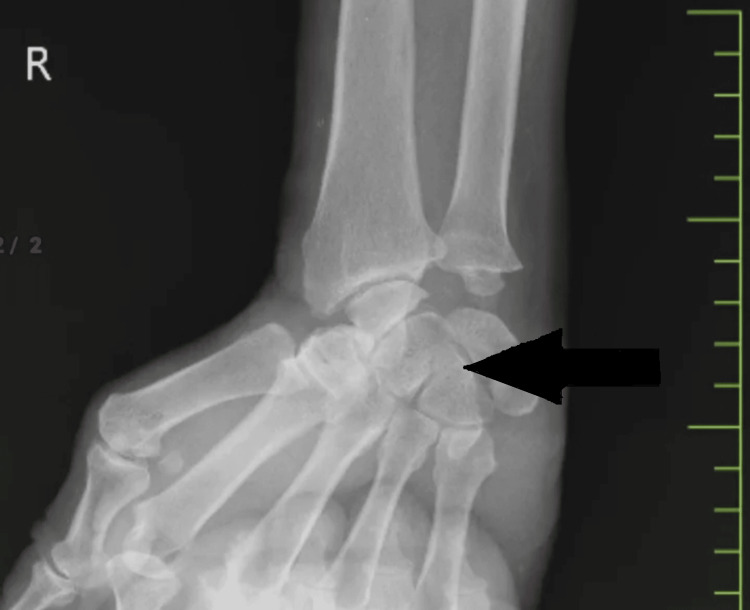
Pre-operative X-ray X-ray showing the right wrist joint. X-ray was taken in antero-posterior view before the operation

**Figure 2:**
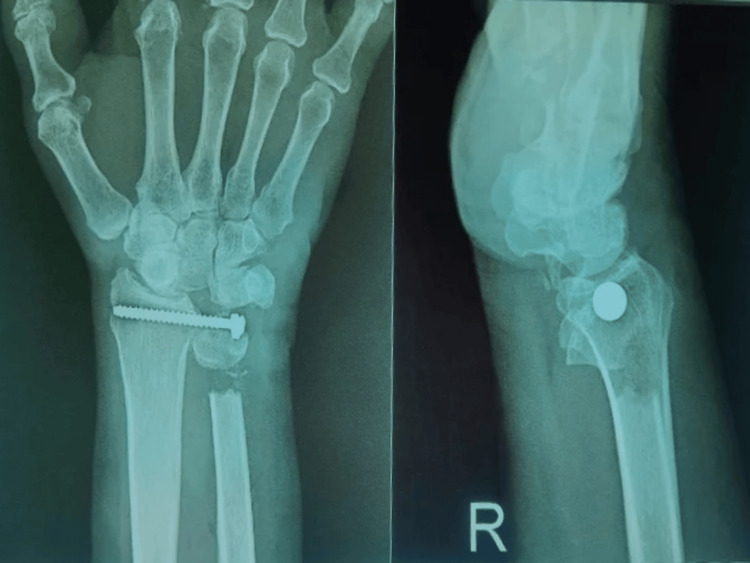
Post-operative X-ray X-ray showing the right wrist joint. X-ray was taken in antero-posterior view and lateral view on 20 August 2023. It shows degenerative changes at the trapezoid and triquetral bones and degeneration and thinning of the triangular fibrocartilage with edema around the styloid process

The patient had a good sense of place, time, and people. On local examination, there was a healthy scar over the volar aspect of the right wrist, which was approximately 4×1 cm. Figure 1 and Figure 2 show the pre-operative and post-operative X-ray images, respectively.

Diagnostic intervention

**Figure 3:**
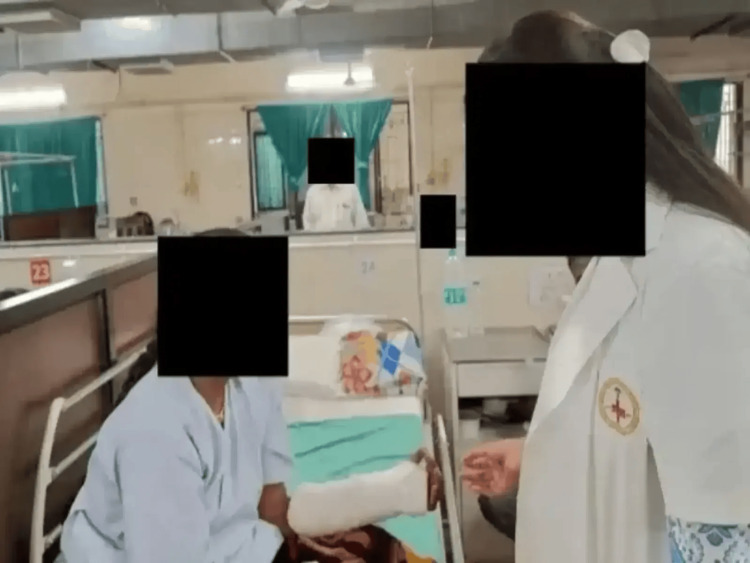
Finger opposition Patient performing opposition movement of the fingers

**Figure 4:**
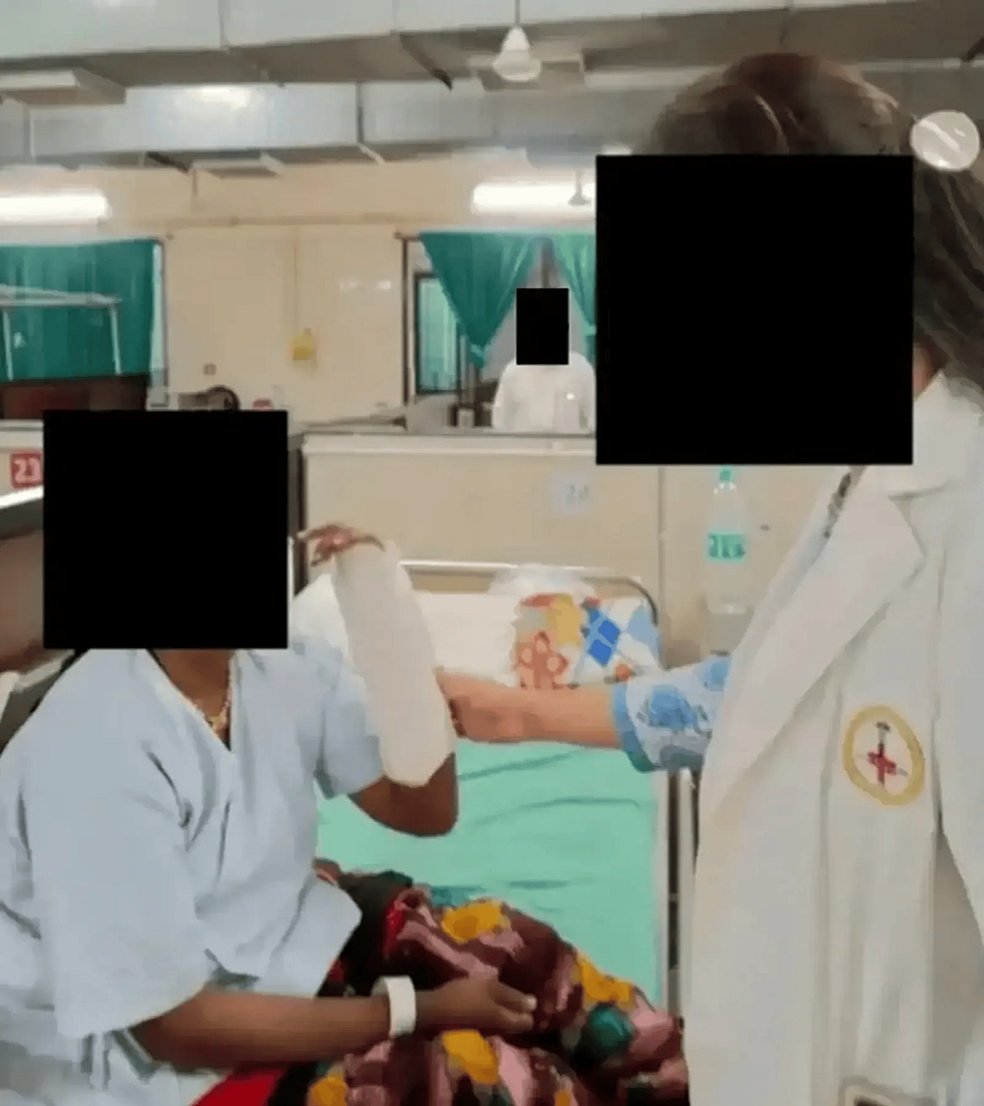
Elbow flexion Patient performing flexion movement of the elbow

**Figure 5:**
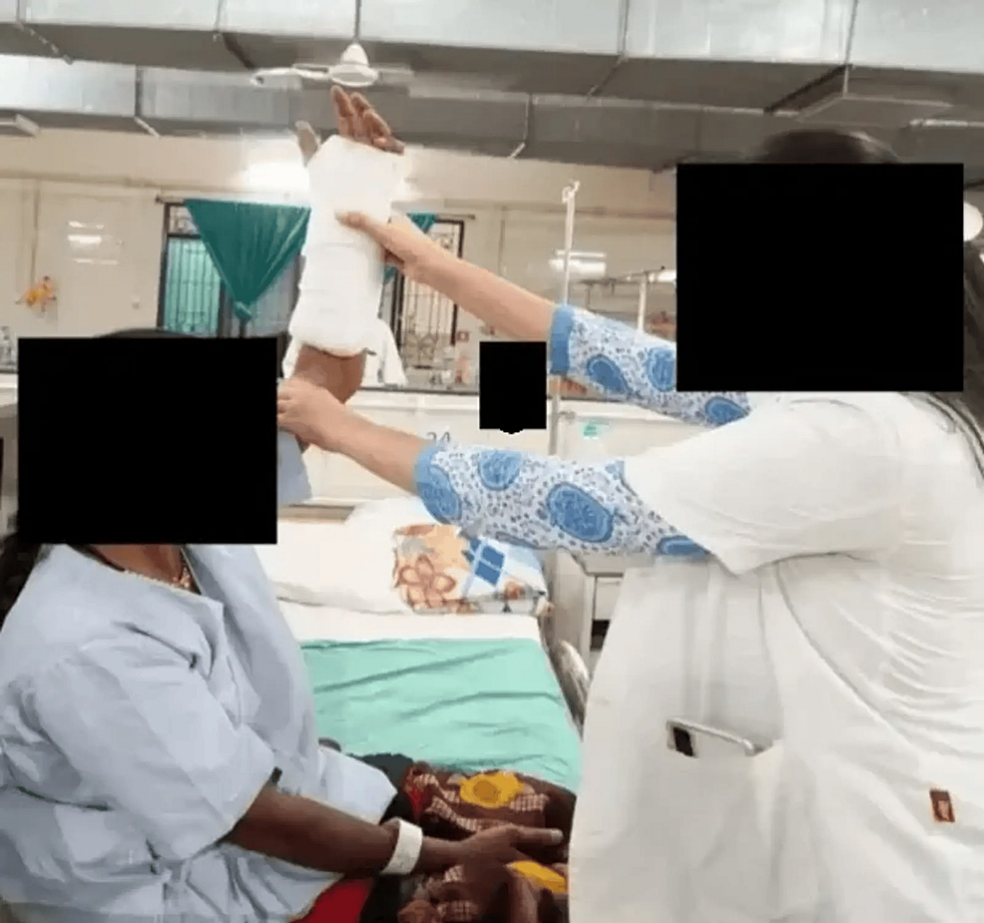
Shoulder flexion Patient performing flexion movement of the shoulder

Figure 3, Figure 4, and Figure 5 show the rehabilitation of the patient.

Physiotherapy rehabilitation

Some goals of the treatment are to educate the patient, reduce her pain, and improve her range of motion, quality of life, and functional ability to do the daily household work.

Table 2 shows the physiotherapeutic protocol.

**Table 2:** Intervention

Goal	Intervention	Rationale
To educate the patient	Lifestyle modifications: to lift less weight with the right wrist, not to lift any heavy weight, and not to do any vigorous activities	To maintain health and increase well-being in life
To minimize pain and swelling	Use cryotherapy and elevate the affected limb	To promote a sense of well-being
To enhance wrist strength	Thera-band wrist exercises	To improve wrist range
To enhance wrist range of motion	Wrist flexion, wrist extension, ulnar deviation, and radial deviation	To improve wrist motion ranges
To improve functional activities of fingers	Opposition exercises	To improve finger mobility
To enhance shoulder range of motion	Shoulder flexion and extension, shoulder shrugs, and shoulder elevation and depression	To improve shoulder mobility and strength
To improve elbow range of motion	Elbow flexion and elbow extension	To improve elbow mobility and strength

## Discussion

Lunate fractures are uncommon and typically coexist with other fractures, ligament disruptions, or dislocations. In order to protect the lunate from shear and compression loads, the therapy entails decompressing it. While some lessen loads, they could put too much pressure on the lunate's ulnar side. Some procedures are extra-articular, protecting the architecture of the mid-carpal joint and the vascularization of the capsule [12]. Following proximal row carpectomy, immediate immobilization is not necessary, and early recovery is crucial. Early rehabilitation could shorten the time needed to regain strength and range of motion, as well as probably the time needed to resume work [13]. A common and effective surgical procedure for post-traumatic and degenerative radiocarpal and intercarpal articular lesions is proximal row carpectomy [14]. Activities for the range of motion were originally started passively and progressed to active-aided activities and then free workouts. There is proof that specialized exercise routines are important for reducing deficits and improving upper-limb function. After several sessions, heating has enhanced range of motion gain that is both acute and sustained in healthy people. In order to encourage motor point and muscle activity in an individual with limited hand function, an electrical muscle stimulant can be employed. Extensor muscle strength and motion range may increase functional abilities [15]. It is debatable whether the carpometacarpal joint should be included in TWA, i.e., total wrist arthrodesis, because difficulties at this location are reported in the literature [16]. When compared to patients with two distal screws, those who had several metacarpal screws had higher rates of plate-related problems and removal, but their post-operative grip strength was unaffected [17]. Patients who did not have fused index and long-ray carpometacarpal joints complained of pain, requiring a second arthrodesis treatment. Patients who perform substantial manual labor should have wrist arthrodesis performed together with index and long finger carpometacarpal joint arthroplasty [18]. Utilizing intramedullary pin fixation, a wrist arthrodesis technique has been enhanced, leading to successful fusion. This technique permits wrist placement at various degrees of ulnar deviation and extension while maintaining the speed and simplicity of earlier techniques [19]. A well-known operation that can be carried out using a variety of approaches is wrist arthrodesis. The most widely acknowledged posture for optimum hand function is between 10° and 30° of extension and 0° and 15° of ulnar deviation [20].

## Conclusions

Proximal row carpectomy and partial carpal fusions serve as standard treatments for wrist arthritis, aiming to preserve some wrist motion while alleviating pain. However, outcomes can be unpredictable. In cases of pancarpal arthritis, total wrist fusion is another established option. The progression from arthrodesis to arthroplasty in other joints, such as the hip, knee, ankle, and shoulder, has influenced the evolving approach to managing wrist arthritis, with advancements contributing to more reliable treatment options. Physiotherapy helps reduce the pain and gain a functional range of motion allowing a good quality of life in such patients.
